# Prevalence and factors associated with post-traumatic stress disorder among survivors of gender-based violence in selected safe houses in the Sidama region, Ethiopia

**DOI:** 10.1007/s44192-026-00445-0

**Published:** 2026-04-16

**Authors:** Mahilet Ashenafi Argaye

**Affiliations:** Department of Psychology, College of Education and Behavioral Sciences, Hawassa University, P. O. Box 05, Hawassa, Ethiopia

**Keywords:** PTSD, GBV, Prevalence, Safe houses, Sidama region, Ethiopia

## Abstract

**Background:**

Post-traumatic stress disorder (PTSD) can significantly affect individuals who have endured traumatic events, particularly in instances of gender-based violence (GBV). This form of trauma can lead to considerable stress that impacts numerous facets of a person's life, including their mental, social, and physical well-being. Recognizing the prevalence of PTSD among survivors of GBV is crucial for understanding the factors that contribute to its development.

**Aims:**

To assess the prevalence of PTSD among survivors living in selected safe houses within the Sidama region of Ethiopia.

**Method:**

A cross-sectional study was conducted involving 84 female survivors of gender-based violence (GBV) from three safe houses. Data were collected using a pre-tested structured questionnaire, which included the PTSD Checklist for DSM-5 (PCL-5).

**Result:**

The findings revealed a PTSD prevalence rate of 66.7% among these survivors (95% confidence interval (CI) 56.4–77.0%). Multivariate logistic regression analysis identified several factors that are significantly associated with an increased likelihood of developing PTSD. Notably, GBV survivors without formal education exhibited an adjusted odds ratio (AOR = 18.62, 95% CI 1.42–24.48) as compared to educated. Additionally, the presence of depressive symptoms was linked (AOR = 10.19, 95% CI 1.15–90.42). Furthermore, severe anxiety symptoms were linked to a higher likelihood of developing PTSD (AOR = 13.77, 95% CI 1.15, 164.74).

**Conclusion:**

These findings highlight the urgent need for effective PTSD treatment for GBV survivors living in safe house within the study region. Improving mental health support services, enhancing the capacities of safe houses, and fostering collaboration with mental health organizations are essential steps to address this critical issue.

## Introduction

### Background of the study

Post-traumatic stress disorder (PTSD) is a mental illness that can affect individuals who have experienced or witnessed a traumatic event. This can include events such as natural disasters, serious accidents, assaults, military combat, or threats of death or sexual violence. Experiencing or witnessing such traumatic events can lead to stress that impacts various aspects of a person's life, including their mental, social, and physical well-being [40]. Flashbacks, nightmares, avoidance, anger, hypervigilance, inattentive, and emotional withdrawal are all possible symptoms of traumatic event in people with PTSD [11]. Furthermore, PTSD has been linked to an increased risk of suicide and suicidal thoughts [13].

Globally, the prevalence of PTSD is approximately 3.9% of the population. However, among individuals who have experienced trauma, this rate increases to 5.6% [19]. The prevalence of PTSD varies significantly across different nations. For example, the lifetime prevalence of PTSD in the general population of the United States is reported to be 8% [8], while in Canada, it is 6.9% [39], and South Korea, it is 1.5% [24].

In developing countries, especially in Africa, the prevalence rates of certain conditions (internal conflict and displacement) are significantly higher. For example, in Kenya, the prevalence is 62.1% [25, 26], while in South Sudan, it stands at 28% [7]. Additionally, the overall prevalence of PTSD in ten sub-Saharan African countries is reported to be 22%, which increases to nearly 30% in regions affected by war or armed conflict [29].

Recent research on adults in Ethiopia has revealed varying incidences of PTSD. The rates identified in different studies are as follows: 67.5% among internally displaced people living in the camps of Debre Berhan [21], 58.4% among individuals affected by inter-communal violence along the border of the Gede'o zone (SNNPR) and the West Guji zone (Oromo region) [20], 40.8% among adult war survivors in Nefas Meewcha Town, located in the South Gondar Zone [36], and 34.5% in war-affected areas of Dessie Town [9]. Currently, there are no studies in Ethiopia linking gender-based violence (GBV) to PTSD. GBV is significant factors in developing PTSD, particularly among women, with studies indicating that up to 50% of those who experience violence also face mental health issues [3]. The relationship is strong, as GBV creates "toxic stress" that can disrupt physiological and immune systems [32].

In the Sidama region, GBV has a prevalence rate of 37.5% [5], yet no studies have assessed PTSD prevalence among GBV survivors in this area. Therefore, this research aims to fill this gap by providing information on the prevalence of PTSD and associated factors among GBV survivors in the Sidama region. Furthermore, it will serve as baseline data for those interested in conducting evidence-based trauma therapies for PTSD in developing countries.

## Materials and methods

### Description of the study area

The study was conducted in safe houses located in Hawassa City and Yirgalem Town, both situated in the Sidama region of Ethiopia. Hawassa, the regional capital, is 275 km south of Addis Ababa, while Yirgalem Town is positioned 315 km south of the capital and 40 km south of Hawassa. In this region, three safe houses provide support for survivors of GBV. These include the Association for Women’s Sanctuary and Development (AWSAD) in Hawassa City and Talita Safe Home, which has branches in both Hawassa City and Yirgalem Town. AWSAD can accommodate up to 70 women and girls who have experienced violence, while Talita Safe Home offers shelter for up to 50 female victims of GBV. Both safe houses deliver comprehensive rehabilitation and reintegration services, encompassing counseling, skills training, and legal support.

### Study population

The study population consisted of individuals from the general community in the Sidama region of Ethiopia.

### Study subject

The source population for this study comprised all survivors of GBV residing in the safe houses of AWSAD and Talita in the Sidama region.

### Inclusion and exclusion criteria

The participants in the studies were women survivors of GBV who were aged 18 and older at the time they attended the safe house. Survivors of GBV, who had linguistic challenges or cognitive impairments, as well as those undergoing severe acute psychotic episodes, were excluded from the study.

### Study design

A descriptive cross-sectional study was used to evaluate PTSD among survivors of GBV in the AWSAD and Talita safe houses located in the Sidama region, from January to June 2025.

#### Sample size and sampling procedure

The sample size was determined using Slovin's formula, based on the following assumptions: a 95% confidence interval (CI) with a Z α/2 value of 1.96, where *p* represents the estimated proportion of the population (since this is unknown, we use 0.5 for maximum variability, due to luck of previous data). The desired precision (d) was set at 0.05 (a 5% margin of error). The population is approximately 120 due to the limited capacity of the safe houses.

$$n = \frac{{\left( {Z_{\alpha /2} } \right)^{2} \left( p \right)\left( {1 - p} \right)}}{{d^{2} }} \times \frac{N}{{N - 1 + \frac{{\left( {Z_{\alpha /2} } \right)^{2} \left( p \right)\left( {1 - p} \right)}}{{d^{2} }}}}$$

A sample size of 92 GBV survivors was taken from safe houses of AWSAD and Talita in the Sidama region, using the Slovin formula mentioned above.

Participants were stratified by safe house and then chosen randomly. Three safe houses were purposefully selected for the research: two located in Hawassa City and one in Yirgalem Town. These locations were chosen due to the availability of active safe houses during the study period and the high number of reported cases of sexual violence against women and girls in the region, as noted by UN Ethiopia [37].

The calculated sample size was 92, which was proportionally allocated based on the maximum capacity of the AWSAD and Talita branches. In the AWSAD branch, there were 70 survivors of GBV, representing 58.3% of the total population. From this branch, 54 survivors participated in the study. The Talita Hawassa branch had 25 GBV survivors; approximately 21% of the total sample, and 19 participants took part from this group. Similarly, the Talita Yirgalem branch also had 25 GBV survivors, accounting for about 21% of the total sample, with 19 survivors participating. Participants from each safe house were selected through simple random sampling from the total number of GBV survivors.

### Study variables

#### Dependent variable

The dependent variable for this study was PTSD.

#### Independent variables

The independent variables for this study include age (18 and above), marital status, religion, education, occupation, depression, and anxiety.

### Measurement tools

PTSD was evaluated using the PTSD Checklist for DSM-5 (PCL-5), developed by Weathers et al. [38]. The PCL-5 is a self-report instrument consisting of 20 items that assess symptoms using a 5-point Likert scale. Here, 0 signifies "Not at all," 1 indicates "A little bit," 2 represents "Moderately," 3 stands for "Quite a bit," and 4 denotes "Extremely." Total scores can range from 0 to 80, with cutoff scores ranges from 31–33 or higher indicating probable PTSD [4, 10]. The original English version of the PCL-5 exhibits strong internal consistency (α = 0.94), excellent test–retest reliability (r = 0.82), and both convergent (rs = 0.74 to 0.85) and discriminant validity (rs = 0.31 to 0.60) [10].

Depression was assessed using the Patient Health Questionnaire (PHQ-9), a self-report tool composed of 9 items. This questionnaire measures depressive symptom on a 4-point scale, where 0 signifies "not at all" and 3 indicates "nearly every day." Total scores can range from 0 to 27, with higher scores reflecting a greater endorsement of depressive symptoms. A score of 10 or above suggests that the respondent may be experiencing depression. The original English version of the PHQ-9 also demonstrates strong psychometric properties, exhibiting high internal consistency (α = 0.86) and robust test–retest reliability (r = 0.82) [2].

Generalized Anxiety Disorder Scale (GAD-7). The GAD-7 is a self-administered questionnaire comprising seven items, each scored on a scale from 0 (not at all) to 4 (nearly every day). This scale evaluates anxiety symptoms based on the DSM-IV-TR criteria [34] within a two-week timeframe prior to the study. Total scores can range from 0 to 21, with cutoff scores of ≥ 5, ≥ 10, and ≥ 15 indicating mild, moderate, and severe levels of anxiety respectively [34]. The original English version of the GAD-7 demonstrates a high internal consistency (α = 0.92) and excellent test–retest reliability (r = 0.83) [34].

All tools were adapted and translated into the local languages of Sidamo Affo and Amharic. Pretesting was carried out on 5% of non-selected samples, leading to necessary adjustments. Cronbach's Alpha was calculated for each tool to evaluate internal consistency. The PCL-5 exhibited a Cronbach's alpha of 0.88, while the PHQ-9 demonstrated an alpha of 0.84, and the GAD-7 received an alpha of 0.82. Each tool achieved calculated scores exceeding 0.70, confirming their reliability. Psychologists conducted interviewer-administered surveys, which were expected to last between 5 to 10 min. Data collectors underwent two days of training, and the principal investigator supervised the data collection process to ensure completeness and adherence to ethical standards.

### Data management and analysis

The data was initially input into Excel and later exported to SPSS Statistics Version 27 for a comprehensive cleaning process aimed at rectifying inconsistencies and addressing missing values before analysis. Descriptive statistics—such as frequencies, percentages, means, and standard deviations—were utilized to characterize the prevalence of PTSD, depression, and anxiety among survivors of gender-based violence (GBV). Bivariate logistic regression analysis was conducted to assess the associations between PTSD with anxiety, depression, and various socio-demographic factors. Independent variables with a significance level of *p* < 0.05 were deemed suitable for multivariable analysis. Subsequently, multivariable logistic regression analysis was performed to control for potential confounders and to identify factors associated with PTSD among GBV survivors, with statistical significance established at *p* < 0.05.

## Result

### Socio-demographic characteristics

A study with a total sample size of 92 participants had 84 active survivors, leading to a response rate of 91.30%. The response rate in this study decreased for several reasons. These factors include clinical issues, such as some participants being unwell, psychological factors, like experiencing low mood during the data collection period, and participants’ leaving the safe house before data collection was completed. The majority of these survivors were aged between 18 and 24 years, accounting for 84.5% of the total sample. Furthermore, a significant portion, specifically 79 individuals (94%), identified as single or not married. In terms of educational qualifications, 70 respondents (83.3%) had completed primary school, while the remaining 14 participants (16.7%) had not received any formal education. Regarding employment, 74 respondents (88.1%) were students, whereas 10 individuals (11.9%) were employed as daily laborers. When evaluating levels of depression, the findings indicated that the majority of participants, specifically 45 respondents (53.6%), experienced moderately severe depression. In terms of general anxiety, most participants reported moderate anxiety, with 37 respondents (44%) affected. Additionally, four individuals (4.8%) experienced minimal anxiety, while the remaining 15 participants (17.9%) were classified as experiencing severe anxiety (see Table 1).

**Table 1:** Sociodemographic characteristics of study participants at AWSAD and Talita Safe houses, Ethiopia, 2025 (n = 84)

Variables	Category	Frequency (N)	Percentage (%)
Age	18–24	71	84.5
	25–34	13	15.5
Marital status	Single	79	94
	Divorced	5	6
Religion	Orthodox	19	22.6
	Muslim	10	11.9
	Protestant	55	65.5
Education	No formal education	14	16.7
	Primary school	70	83.3
Occupation	Student	74	88.1
	Daily laborer	10	11.9
Depression	Mild	13	15.5
	Moderate	26	31
	Moderately Severe	45	53.6
Anxiety	Minimal anxiety	4	4.8
	Mild Anxiety	28	33.3
	Moderate Anxiety	37	44
	Severe Anxiety	15	17.9

### Prevalence of PTSD

The overall prevalence of PTSD among survivors of gender-based violence (GBV) was found to be 66.7% (95% CI 56.4–77.0%). Among survivors aged 18 to 24, a significant majority, 46 individuals (64%), displayed symptoms of PTSD. In the 25 to 34 age group, the prevalence was even higher, with 10 survivors reporting a rate of 76.9%. Examining marital status, a substantial proportion of single survivors—52 individuals (65.8%)—demonstrated signs of PTSD. In another marital category, four out of five survivors (80%) also reported PTSD symptoms. Regarding occupational status, 48 students (64.9%) indicated they had experienced PTSD, while 8 daily laborers (80%) similarly developed the condition. Additionally, a clear correlation was observed; as levels of depression and anxiety increased, so did the likelihood of developing PTSD (See Table 2).

**Table 2:** Proportion of PTSD among the survivors at AWSAD and Talita Safe houses, Ethiopia, 2025 (n = 84)

Variables	Category	Post-traumatic stress disorder	
		Yes N (%)	No N (%)
Age	18–24	46(64.8)	25(35.2)
	25–34	10(76.9)	3(23.1)
Marital status	Single	52(65.8)	27(34.2)
	Divorced	4(80)	1(20)
Religion	Orthodox	9(47.4)	10(52.6)
	Muslim	8(80)	2(20)
	Protestant	43(78.2)	12(21.8)
Education	No formal education	13(92.9)	1(7.1)
	Primary school	43(61.4)	27(38.6)
Occupation	Student	48(64.9)	26(35.1)
	Daily laborer	8(80)	2(20)
Depression	Mild	8(61.5)	5(38.5)
	Moderate	21(80.8)	5(19.2)
	Moderately Severe	27(60)	18(40)
Anxiety	Minimal anxiety	0(0)	4(100)
	Mild Anxiety	14(50)	14(50)
	Moderate Anxiety	29(78.4)	8(21.6)
	Severe Anxiety	13(86.7)	2(13.3)

### Factors associated with PTSD

A multiple logistic regression model was utilized to identify the factors associated with PTSD. In this model, β0 represents the constant term, while β1, β2, β3, and so on up to β8 are the regression coefficients for the independent variables X1 through X8. These variables include age, marital status, education level, occupation, depression, and anxiety.

The Omnibus tests of model coefficients produced a χ2 value of 43.016 with a *p*-value of less than 0.001, indicating a highly significant result. This finding suggests that the predictor variables listed in Table 3 collectively play a significant role in predicting the occurrence of PTSD. The model’s chi-square value was 57.493 on 8 degrees of freedom, which was also highly significant at levels beyond 0.005. This indicates that including the explanatory variables significantly improved the fit of the full model compared to the constant-only model. The Cox and Snell and Nagelkerke pseudo R-squared values for the model were 0.401 and 0.574, respectively. Additionally, the Hosmer–Lemeshow test produced a chi-square value of 2.957 with a *p*-value of 0.937 on 8 degrees of freedom. Since this *p*-value exceeds both the 0.01 and 0.05 thresholds, it suggests there is no significant difference between the observed values and those predicted by the model, indicating that the model adequately fits the data. Furthermore, the sensitivity and specificity analysis revealed that 85.7% of PTSD cases were accurately predicted within their respective categories.

**Table 3:** Factors associated with PTSD among the GBV survivors at AWSAD and Talita Safe houses, Ethiopia, 2025 (n = 84)

Variables	Category	PTSD			
		Yes N (%)	No N (%)	COR (95% CI)	AOR (95% CI)
Age	18–24	46(64.8)	25(35.2)	1	1
	25–34	10(76.9)	3(23.1)	1.81(0.46–7.19)	1.54(0.25–9.49)
Marital status	Single	52(65.8)	27(34.2)	0.48(0.05–4.52)	1.39(0.07–30.06)
	Divorced	4(80)	1(20)	1	
Education	No formal education	13(92.9)	1(7.1)	0.12(0.02–0.99)*	18.62(1.42–24.48)*
	Primary school	43(61.4)	27(38.6)	1	1
Occupation	Student	48(64.9)	26(35.1)	1	
	Daily laborer	8(80)	2(20)	2.17(0.43–10.97)	2.68(0.21–33.64)
Depression	Mild	8(61.5)	5(38.5)	1	1
	Moderate	21(80.8)	5(19.2)	2.63(0.60–11.57)	10.19(1.15–90.42)*
	Moderately Severe	27(60)	18(40)	0.94(0.3–3.79))	1.49(0.27–8.30)
Anxiety	Minimal anxiety	0(0)	4(100)	1	1
	Mild Anxiety	14(50)	14(50)	1.14(0.21–6.16)	1.55(0.19–12.38)
	Moderate Anxiety	29(78.4)	8(21.6)	4.67(0.86–25.31)	8.11(0.96–68.67)
	Severe Anxiety	13(86.7)	2(13.3)	8.67(1.05–71.57)*	13.77(1.15–164.74)*

*Significant association at *p* < 0.05

The analysis revealed a significant correlation between PTSD and variables such as level of education, depression, and anxiety. Notably, those who had no formal education were 19 times more likely to experience PTSD (Adjusted Odds Ratio [AOR]: 18.62, 95% Confidence Interval [CI] [1.42, 24.48], *p* < 0.05) than those who attended primary education. Regarding depression, respondents who developed moderate depression were 10 times more likely (AOR: 10.19, 95% CI [1.15, 90.42], *p* < 0.05) to experience PTSD compared to those who developed mild depression. Furthermore, participants who had severe anxiety were 14 times more likely to experience PTSD (AOR: 13.77, 95% CI [1.15, 164.74], *p* < 0.05) than those who had minimal Anxiety (Table 3).

## Discussion

The prevalence of PTSD was 66.7% in safe houses, indicating a significantly high occurrence of PTSD in the study area. These findings are in line with those reported by Makango et al. [21] in Debre Berhan, Ethiopia, which recorded a prevalence of 67.5%. Similarly, comparable rates have been observed by Taru et al. [35] in North Eastern Nigeria (63%), Musau [25, 26] in Kenya (62.1%), and Madoro et al. [20] in both the Gede'o zone of SNNPR and the West Guji zone of the Oromo region in Ethiopia (58.4%). However, the prevalence rate found in this study is notably higher than those in several other studies conducted in Ethiopia, which reported rates of 34.5% in Dessie [9], and 30.7% in Dabat Melese et al. [23]. It also exceeds the rates reported in other African countries and regions, such as South Sudan (28%) [31], Libya (25.23%) [1], South Africa (21%) [28], and Uganda (18%) [18]. The Discrepancies in prevalence rates may arise from the varying populations studied. In this particular research, the sample was drawn from safe houses specifically designated for survivors of GBV. The findings revealed that a significant percentage of these individuals develop PTSD, with rates ranging from 55 to 92% [14, 16]. These figures are markedly higher than those observed in the general population, where the prevalence for women is approximately 10.4% [27].

The World Health Organization (WHO) estimates that 15–20% of survivors of GBV may experience mild to moderate mental health disorders (depression and anxiety) [41]. However, in the current study, over half of the participants reported symptoms of moderate severe depression and anxiety. This indicates a high prevalence of mental health issues among women who have experienced GBV. These findings align with the report by Hossain et al. [15] from Kenya.

The results of the current study showed that Study participants who had no formal education were 19 times more likely to experience PTSD than those who attended primary education. This result was consistent with previous studies reported that educational level is associated with PTSD with individuals with higher levels of education less likely to develop PTSD [36, 42]. Furthermore, research shows that higher educational attainment is associated with a reduced prevalence of PTSD and better mental health outcomes, acting as a protective factor [12].

Concerning depression, respondents who developed moderate depression were ten times more likely to experience PTSD compared to those who developed mild depression. This study aligns with the findings of Nathanson et al. [27], which indicate that depression and PTSD often occur together as significant comorbidities. Similarly, participants with severe anxiety were more likely to develop PTSD compared to those with minimal anxiety (Table 3). This finding aligns with the report by Isaac et al. [17], which states that individuals exhibiting high levels of severe anxiety have a significantly greater likelihood of developing PTSD following a traumatic event than those with lower levels of anxiety.

Finally, the current study found that individuals with severe depression and anxiety tend to develop more PTSD compared to those with mild symptoms. This finding aligns with previous research on veterans in the King County Jail system in Seattle, as well as studies conducted in Kenya and Ethiopia [6, 22]. This could be attributed to the fact that participants experiencing depressive and anxiety symptoms are more likely to develop PTSD than those without such symptoms [30, 33].

## Conclusion and recommendations

The current study reveals a significant prevalence of PTSD among survivors of GBV residing in selected safe houses in the Sidama region of Ethiopia. Importantly, factors such as education level, depression, and anxiety have been identified as key predictors of PTSD. These findings have serious implications, emphasizing the need for an integrated healthcare approach to address the multifaceted needs of these individuals. There is an urgent requirement for effective PTSD treatments, such as prolonged exposure therapy (PE), specifically tailored for GBV survivors in safe houses within the region. Consequently, It is essential to enhance mental health support services, increase the capacity of safe houses, and foster partnerships with mental health organizations by collaborating with key stakeholders, including regional health bureaus, NGOs, and mental health service providers. Additionally, there should be an increased focus on research that explores a wider array of variables.

### Strengths and limitations

This study is significant as it represents one of the initial efforts to explore the socio-demographic factors associated with PTSD among survivors of GBV residing in safe houses within the Sidama region of Ethiopia. However, several limitations should be recognized. First, this cross-sectional study examines a limited number of victims with a limited carrying capacity of the victims of operational safe houses in the region. Second, the absence of previously validated Amharic versions of the questionnaires has constrained the range of variables available for analysis. Overall, to avoid overgeneralization, the conclusions should be regarded as preliminary or exploratory.

## Data Availability

The data are available at special request from the corresponding author.
